# Supplementary material for: Are carbon emissions trading and green financial instruments synergistic? -Comprehensive quantitative research based on content analysis
Source: PLoS One. 2024 Mar 7;19(3):e0298601. doi: 10.1371/journal.pone.0298601 (PMC10919601; doi:10.1371/journal.pone.0298601)
Supplement: S1 Data — (ZIP) [file pone.0298601.s001.zip › Data/地级市Z_F工作报告.zip!/╣π╢1⁄2/╓╨╔╜╩╨/└┤╫╘╣1⁄2╓┌║┼í╛┬φ┐╦╩2╛▌═°í┐_2006.docx]

各位代表：

我代表市人民政府向大会作政府工作报告，请予审议，并请政协委员和其他列席人员提出意见。

“十五”时期及2005年工作回顾

“十五”时期是我市现代化建设取得新的重大成就的五年。五年来，在省委、省政府和市委的正确领导下，我市高举邓小平理论和“三个代表”重要思想伟大旗帜，深入学习贯彻党的十六大，十六届三中、四中、五中全会和胡锦涛总书记视察中山重要讲话精神，牢固树立和落实科学发展观，大力推进和谐社会建设，坚持“工业强市”、“依法治市”、“科教兴市”和“可持续发展”战略，积极贯彻落实国家的宏观调控政策，迎难而上，保持了全市经济社会稳健发展；成功战胜非典型肺炎和百年一遇特大洪灾的袭击。市十二届人大二次会议确定的预期目标已经实现，市十一届人大三次会议通过的“十五”计划确定的各项任务全面完成，标志着我市全面建设小康社会、率先基本实现社会主义现代化的进程迈入新的发展阶段。

经济总体实力显著增强。“十五”时期全市生产总值年均增长18.1%，超过“十五”计划预期目标7.6个百分点。2005年全市实现生产总值817.6亿元，人均7200多美元，分别是“九五”期末的2.4倍和2.2倍；2005年地方财政一般预算收入、全社会固定资产投资、社会消费品零售总额分别达到54.3亿、321.4亿和276.6亿元，分别比“九五”期末增长210.3%、192.3%和95.1%。民营经济迅速发展，“十五”期间新增民营企业共9.4万家。对外贸易规模扩大，质量提高，2005年进出口总额是2000年的3倍多。五年累计实际利用外资26.6亿美元。人民生活水平不断提高，2005年城镇居民人均可支配收入比2000年增长45.3%。

产业结构进一步优化。承接国际产业转移的大趋势，推进产业强市战略，第二产业成为经济发展的支柱，三次产业比例由2000年的6.8:52.4:40.8调整为2005年的3.8:61.4:34.8。产业集群快速壮大，拥有国家级产业基地19个，省级专业镇10个。积极推进名牌战略，荣获“全国品牌经济城市”称号，拥有国家级、省级名牌、名标262个。

“三农”工作取得新成效。农业基础地位得到加强，综合生产能力不断提高。主要农作物优良品种覆盖率达98%以上，农业效益稳步提高。农业产业化、标准化进程加快，农业科技贡献率达59%。基础设施建设水平不断提高，“十五”期间投入超过10亿元用于“农村五项工程”建设，新建4米以上农路1528公里，农桥824座，涵闸242座，整治低产渔塘20万亩，整治内河涌735公里，完成“一区三线”林相改造工程4.4万亩，“四旁”绿化1946公里，投资6.1亿元建设了201宗城乡水利防灾减灾工程。三年来共转移农村富余劳动力就业6.4万人，推进农村税费改革，全面免征农业税，农民人均年纯收入比“九五”期末增长66%。农村股份合作制改革全面推进。村级财务管理和村务公开等各项管理制度逐步完善。

各项改革深入推进。国有资产运营监督管理体制不断完善，市属国有企业整体实力和市场竞争力逐步增强。顺利完成新一轮政府机构改革。公务员收入分配制度、公务用车、行政审批制度以及人事管理制度改革成效显著，行政审批事项减幅达75.1%。计划、投融资、财税、流通、金融、农村经济体制和住房、医疗制度改革全面推进，技术、劳动力等要素市场发展加快，市场配置资源的基础性作用进一步增强。加快构建公共财政框架。科教文卫各项事业管理体制改革取得新成绩。

基础设施建设取得新进展。“十五”期间，全市共投入57.9亿元用于基础设施建设，全市供电总量431.1亿千瓦时，较“九五”时期增长115.8%，积极探索BOT等项目运作方式，投融资渠道不断拓宽。一大批重点工程完工，城市承载力不断增强。全市公路总里程达到1531公里。坚持公交优先战略，公交站场、线路建设有新进展，完成了一批城区道路改造，城市交通网络日益完善，“畅通工程”、“智能交通系统工程”通过验收。全市信息化指数达到78%以上。组团发展战略逐步推进，东部组团发展率先启动。

各项社会事业蓬勃发展。在全市上下的共同努力下，我市荣获“全国文明城市”称号，以各项创建活动为载体，全面贯彻和实施《公民道德建设实施纲要》，“博爱、创新、包容、和谐”的新时期中山人精神得到进一步弘扬，市民文明素质得到进一步提高。教育事业加快发展，成功创建省“教育强市”。加强自主创新能力建设，拥有国家级、省级工程技术研究开发中心10家。劳动和社会保障事业发展迅速，积极推进农村基本养老保险工作。推动镇村医疗卫生一体化，公共卫生体系不断完善，新型农村合作医疗覆盖面达96.2%。

2005年是我市全面落实科学发展观、改革开放和现代化建设取得新成绩的一年。一年来，市政府按照省委、省政府和市委的工作部署，抓落实、重提高、促发展，努力克服多种困难，实现了全市经济既快又好的发展，主要体现在以下几个方面：

加大产业结构调整力度，全面提升产业竞争力。实现工业总产值2495.7亿元，增长21.7%，工业增加值473.9亿元，增长15.5%，工业对经济增长贡献率为59.7%，规模以上工业企业经济效益综合指数为130.9%。产业高级化、适度重型化趋势增强，装备制造业（规模以上）产值520.9亿元，增长18.2%。高新技术产品产值600亿元，增长31%。五大支柱产业带动作用突出，生物医药、汽车配件、包装印刷等特色产业有较大增长，产业集群优势不断增强。大力推进工业技改，共投入3623万元推动企业实施技改99项，带动投资27.4亿元。东升、板芙成为广东省专业镇技术创新试点。新增中国名牌产品3个，中国驰名商标1个，国家免检产品27个。积极推动产业转移，与阳西合作创建了产业转移园区。积极盘活闲置土地，土地集约利用效率进一步提高。推进节约型社会建设，每万元生产总值综合能耗下降0.3%。

加快农业“三化”进程，农村经济稳步发展。实现农业总产值52.2亿元，增长2.5%。效益农业、特色农业、品牌农业和绿色农业取得新发展，省级农业名牌产品增至22个，国家绿色食品认证产品增至9个。拥有省、市两级农业龙头企业27家，农业标准化生产基地19个。农业科研攻关和技术推广应用效果显著。市级财政共投入4700万元用于“农村五项工程”建设。启动83宗城乡水利防灾减灾工程建设。粮食种植面积和产量超额完成省下达的任务。动物疫病及有害生物防控工作成效显著。160个村完成农村股份合作制改革，农村低保水平进一步提高。基层建设得到加强，农村社会稳定。

统筹规划建设，改善生态环境。做好新一轮城市总体规划纲要编制工作。全社会固定资产投资321.4亿元，增长10.3%，重大项目建设进展顺利。交通基础设施建设跃上新台阶。中江高速公路、西部沿海高速公路、南外环城南路以东段、城桂公路二期建成通车，博爱路和起湾道改造完工。整治危桥126座，渡口8个。能源项目和电网建设加快，完成天然气管道主工程。涉及民生的重点项目加快推进，完成中心组团垃圾综合处理基地垃圾焚烧发电厂、大涌口水闸、田基沙水闸、文化艺术中心、中山高级职业技术学院等建设工程。全面启动生态市创建工作，设立五桂山生态保护区，清洁生产、治污保洁和生态保护工作进一步加强，被授予“国家级生态示范区”称号。大力开展创建“全国环境优美乡镇”工作。推进新一轮“绿化中山大地工程”，中心城区绿化覆盖率达39.4%。规范土地市场。加强城管综合执法，城市管理上新水平。

重视内外源经济发展，提高经济自主增长能力。民营经济加快发展，规模以上民营工业企业实现产值678.9亿元，增长19.9%。服务业健康发展，实现增加值284.9亿元，增长16.6%。金融、证券、保险业运行良好。积极开拓“两个市场”，实现外贸进出口总值186.9亿美元，增长19.7%。加大招商引资力度，实际利用外资6.5亿美元，增长28.3%。新增外商直接投资企业320家，外资企业增资扩产总额4.7亿美元。成功举办3·28招商经贸洽谈会等系列会展。积极参加粤台经济技术贸易交流会等活动。实施“走出去”战略，全市对外直接投资2300多万元。

金融、财税稳健发展，人民生活改善。深化投融资体制改革。市级单位全部实行部门预算，继续推进国库管理制度改革扩面，加强和规范非税收入征收，完善政府采购工作。农信社改革全面铺开。大力整顿和规范市场经济秩序，打击传销、制假售假，食品安全及农产品安全等专项行动成效显著。“诚信中山”建设不断推进。城乡居民生活水平不断提高，城镇居民人均可支配收入17255元，增长9%；农民人均年纯收入8102元，增长10.1%。城乡就业工作成效显著，城镇登记失业率为2.1%。社会保障体系不断完善，全市参保达349.4万人次，养老、失业、工伤、生育待遇水平逐步提高。农村基本养老保险工作顺利推进，农民参保达22.1万人。完善城乡孤寡老人集中供养、城乡最低生活保障制度，推动农村敬老院建设。切实关注社会困难群体，扶困助学基金总额达1220万元，帮助6700位贫困家庭子女入学。完成新一届村（居）委会选举工作。信访工作得到加强，群众集体访批次和人次分别同比下降34.8%和46.5%。推行预存工资保障制度，解决建设领域拖欠工程款取得新进展。加强社会治安综合治理，加强流动人口和出租屋管理工作，获得全国社会治安综合治理优秀地市“长安杯”。落实措施，加强安全生产工作。

社会事业跃上新台阶。大力推进“科教兴市”战略的实施，全市共有教育强镇19个。振兴初中行动计划初见成效，20个镇（区）共新建、扩建初中54所。启动中学优质化工程，中小学优质学位覆盖率超过80%。全市高考录取率达93.8%。民办教育、职业技术教育、成人教育加快发展。继续完善人才“柔性流动”和“绿色通道”政策，全市人才总量不断增加。成为省知识产权试点城市，5家企业获国家火炬计划重点高新技术企业称号。成功举办博鳌亚洲论坛2005国际文化产业会议，市图书馆成为国家一级图书馆。不断完善公共卫生服务网络，爱国卫生运动深入推进，有国家级、省级卫生镇15个。切实做好猪链球菌、禽流感等动物传染病防控工作。全面完成省下达的人口计划生育任务，全市户籍人口出生率为10.8‰，计划生育率为93%。获得“全国全民健身样板城市”称号，成功承办2005年全国田径大奖系列赛中山站赛事，市、镇、村三级社区体育设施网络进一步完善。

政府自身建设进一步加强。深入贯彻落实《行政许可法》和《全面推进依法行政实施纲要》，成立市政府法律顾问室。自觉接受市人大及其常委会的法律监督和市政协的民主监督，重大问题征求人大代表、政协委员意见。切实改进工作方式，通过约请面谈、组织视察、市政府领导亲自办理重点议案、提案等多种方式，拓宽有效监督渠道，完善“人大代表建议和政协提案网络办理在线系统”，办理人大代表议案、建议139件、政协委员提案176件，答复率为100%，满意率不断提高。政府职能转变进一步深化，社会管理和依法行政工作取得新成效。推进行政管理体制改革，设立五桂山街道办事处，推进火炬区与南朗镇资源整合。行政审批制度改革继续推进，全市31个部门的157个审批事项实现网上办理。加大政府督查及投诉处理力度。坚持政府新闻发布和重大事项听证制度。扎实开展纠风工作，加强电子政务建设，政务公开渠道进一步拓宽。

民政、监察、外事、侨务、台务、审计、统计、老干、广播电视新闻出版、气象、国防建设、民兵预备役、档案、地方志、妇女儿童、残疾人、民族宗教、人防、老区建设等各项工作取得了新的成绩。

各位代表，“十五”时期是我市经历挑战、经受考验最多的时期之一，也是经济社会发展最快、城乡面貌变化最大、老百姓得到实惠最多的时期之一。“十五”时期的工作成绩来之不易。这是省委、省政府和市委正确领导的结果，是市人大及其常委会依法监督，市政协以及各民主党派民主监督和支持的结果，是全市人民群众努力奋斗的结果。我谨代表市政府向全市广大工人、农民、知识分子、干部职工、驻我市人民解放军指战员、武警官兵、人民警察和各界人士表示崇高的敬意！向长期关心支持我市建设的港澳台同胞、海外侨胞和国内外友人表示衷心的感谢！

我们要清醒地看到，当前经济社会发展正进入转型期，发展中还存在许多突出的矛盾和问题：经济结构不够合理，增长方式比较粗放，资源环境压力加大；区域发展不平衡；自主创新能力不强，发展后劲不足；社会稳定、安全生产管理等还存在隐忧和薄弱环节；按照新形势和科学发展的新要求，政府职能转变还不能完全适应市场经济要求等。这些问题都需要在今后的工作中切实加以解决。

“十一五”展望及2006年工作安排

“十一五”时期是我市进一步落实科学发展观，建设“两个适宜”城市，构建“和谐中山”，全面建设小康社会、率先基本实现社会主义现代化的关键时期。从国际环境看，全球化趋势进一步发展，国际资本流动加快，中国-东盟自由贸易区建立，科技进步为我们在技术跨越的基础上实现经济的跨越式发展提供了现实可能；从国内环境看，结构调整和改革开放的效果持续释放，经济自主增长的内在机制初步形成，居民消费结构升级、城市建设步伐加快等发展趋势明显，为继续保持较快增长创造了良好的环境；从区域发展环境看，泛珠三角经济合作进一步加强，珠三角城市群正不断推进协调与合作；从我市内部环境看，经过多年的发展，积累了一定的经济实力、产业基础和成功经验，市场经济体制逐步完善，经济发展能力不断增强，一系列针对经济转型的政策、措施正逐步发挥效应，全市经济保持健康稳步发展势头，为实现可持续发展和再上新台阶奠定了坚实的基础，未来五年的发展大有可为。同时，也必须清醒地看到，今后的发展仍面临国际石油价格高企和贸易保护主义加剧，城市间的竞争日趋激烈，经济发展中生产要素制约日益明显等许多困难。全市上下要增强危机意识、责任意识和发展意识，在经济社会转型的关键时期，真正把科学发展观的要求落实到建设和发展的各项具体工作中，立足当前，着眼长远，坚持“加快发展、率先发展和协调发展”，创新发展理念和发展模式，推动经济社会切实转入科学发展的轨道，不断实现新的跨越。

“十一五”时期政府工作的总体要求是：以邓小平理论和“三个代表”重要思想为指导，认真贯彻党的十六届三中、四中、五中全会和省委九届七次、八次全会精神及市委十一届三次全会精神，以科学发展观统领经济社会发展全局，以建设“两个适宜”城市，构建“和谐中山”，实现富裕安康为总目标、总任务，坚持抓好发展第一要务；不断深化改革，扩大开放，加快经济增长方式转变；增强自主创新能力，推进节约型社会建设；统筹城乡发展，建设社会主义新农村；协调社会利益关系，维护社会和谐稳定，为建设富裕型小康社会、率先基本实现社会主义现代化打下更加坚实的基础。综合考虑未来五年我市发展的趋势和条件，具体要实现以下几方面目标：

——产业竞争力增强。产业结构进一步优化，经济增长的质量和效益明显提升。农业产业化水平明显提高，高新技术产业、装备制造业和服务业加快发展；全市生产总值年均增长11%，2010年，人均生产总值超过1万美元。

——城乡协调发展。努力建设社会主义新农村，统筹城乡基础设施建设，加快农村社会事业发展，进一步凸显四大组团的辐射带动作用，东部沿海在我市经济发展中的龙头和战略地位更加明显，逐步形成功能互补、布局合理、协调互动的新型城乡关系。2010年城市化率达到78%。

——社会文明进步。大力发展文化产业。科技、教育、文化、卫生、体育等多项事业全面加快发展，市民素质明显提高，民主法制建设和精神文明建设取得新进展。到2010年，高中阶段教育毛入学率达到85%，高等教育毛入学率达到50%；加强人口与计划生育工作，居民平均期望寿命达76.5岁，基本实现教育和医疗卫生事业现代化。

——环境优美和谐。积极创建国家生态市。到2010年，城镇生活污水处理率提高到70%，城镇生活垃圾无害化处理率达到80%以上；城镇居民人均公共绿地面积提高到12平方米；循环经济、清洁生产和清洁能源推广取得新成效，2010年万元生产总值能耗比“十五”期末下降13%。

——体制健全高效。社会主义市场经济体制进一步完善，初步形成高效透明、运转协调、行为规范的行政管理体制；基本完成社会领域和公共事业领域政企分开、政事分开、管办分开的改革；农村经济体制、投融资体制、公共财政体制等各个领域的改革取得新进展。

——人民富裕安康。人民生活水平和生活质量进一步提高。到2010年，农民人均年纯收入年均增长6%，城镇居民人均可支配收入年均增长5%；社会保险总参保达510万人次以上；城镇登记失业率控制在3%以内；公共安全和应急救援机制进一步完善，处置突发事件能力明显增强。

2006年是实施“十一五”规划的第一年，是站在新的起点谋划未来、建设富裕型小康社会的关键一年，做好2006年的工作意义重大。根据《中山市国民经济和社会发展第十一个五年规划纲要》的要求，2006年全市经济和社会发展的预期目标为：全市生产总值增长12%；工业总产值增长15%；工业增加值增长13.5%；全社会固定资产投资增长8%；社会消费品零售总额增长13%；外贸出口增长12%；城镇居民人均可支配收入增长8%，农民人均年纯收入增长8%；城镇登记失业率控制在3%以内；人口自然增长率控制在6.5‰以下。为实现上述目标，必须着力抓好以下工作：

一、增强自主创新能力，加快优化经济结构和提升产业素质

推动优势产业整合和集聚，向高级化和适度重型化方向发展。加快产业转型升级，建立现代产业体系，是我市经济发展的迫切需要。要制定和完善产业发展规划和配套政策，安排1.19亿元用于技改贴息、扶持装备制造业、中小企业发展和推进名牌战略。发挥19个国家级制造业生产基地的“集群制造”优势，建设产业集群升级示范区，依托工业园区、专业镇、特色产业基地，推进产业结构从“中山制造”向“中山创造”的战略性转型。继续推动装备制造业发展，重点发展电子及通信设备制造业和通用设备制造业，培育龙头型企业，加强基地建设。做大医药、新材料、新型环保、光机电一体化、精细化工和包装印刷等具有较大潜力的产业。以国家健康科技产业基地为依托，大力发展中医药产业。运用高新技术和先进适用技术推动电气机械、金属制品、纺织服装、食品等传统产业优化升级。大力发展临港工业。全面推进开发区和产业聚集区建设，优先保障重大项目建设的用地需求，重点发展技术、资本密集型产业和现代服务业。加强工业项目投资强度、产出强度、单位产值生产能耗和税收贡献率考核。

加强自主创新能力建设，大力发展品牌经济。完善自主创新的政策和市场环境，加快建设以企业为主体的创新体系，构建多元化服务体系。重点抓好引进、消化和吸收，加快发展具有自主知识产权、核心技术的基础性产业和增长性战略产业。通过“品牌输出，产业转移，拓宽空间”，加快推进和实施“总部战略”，鼓励企业低成本扩张，建设产业转移工业园。实施名牌带动战略，积极培育拳头产品。打造区域品牌，提升产业集群的品牌经营水平和国际竞争力。

激发民间资本活力，促进内源型经济由小到大转变。放宽民营资本准入范围，鼓励社会机构设立信用担保基金和外资银行进入，推动优质企业上市融资。加快建设技术创新平台和服务体系，培育一批扎根本土、具有自主品牌、核心技术和国际竞争力的“土根企业”，形成自主、内生的经济增长动力，加快民营科技园建设。鼓励民营企业加快建立现代企业制度，培育和造就更多的优秀企业和优秀企业家，提升民营企业的整体素质和竞争力。

二、全面加快“三化”进程，建设社会主义新农村

提高农业综合生产能力，加快现代农业发展。按照生产发展、生活宽裕、乡风文明、村容整洁、管理民主的要求，全面推进社会主义新农村建设。要进一步完善和强化对农业的支持政策。继续推进农业产业结构调整，增强科技对农业的支撑作用，全面提高农业综合生产能力。推进农业产业化经营和标准化生产，引导农业向适度规模化发展。大力培育农业品牌，扩大养殖、园艺等劳动密集型产品和绿色、无公害食品的生产，发展高产、优质、高效、生态、安全农业，加快现代农业园区、示范区建设。加强对耕地特别是基本农田的保护，确保完成全年粮食任务。推进城乡水利工程建设，投入4.3亿元重建20座水闸（泵站），启动西河水闸重建工程，进一步提高防灾减灾能力。健全农产品质量管理体系，加强动植物防疫检疫工作。

全面推进农村各项改革，千方百计增加农民收入。鼓励农民自主创业和农村富余劳动力转移就业，探索建立城乡一体化的劳动力失业登记管理制度。完善农村产权管理体制，建立城乡统一、适应市场经济发展的产权管理制度。继续深化农村金融体制改革。加快推进农村股份合作制，壮大农村集体经济。完善集体土地管理体制，依法保障农民在土地流转中产生的增值收益。积极化解镇村集体债务。

统筹城乡发展，加快城镇化进程。着力推进城市基础设施向农村延伸，城市社会服务事业向农村覆盖，城市文明向农村辐射。扩大公共财政覆盖农村的范围，确保新增社会保障、教育、文化、卫生、环保等事业经费向农村倾斜，逐步在农村普及图书室、健身场所、休闲公园等设施。推进“农村五项工程”，完善农村电网、通讯等设施的建设。加快“村改居”、“城中村”改造。严控农村宅基地审批，以农民居住小区建设为切入点，加快中心村和中心镇建设，提高城市化水平。加强农村资源综合利用，加快供水、供气、污水处理、垃圾处理等设施建设。全面推进创建“全国环境优美乡镇”活动。

三、大力发展现代服务业，推动外经贸增长方式转变

充分释放服务业发展潜力。贯彻《关于加快我市现代服务业发展的意见》，进一步推进服务管理体制创新，促进服务业与制造业融合发展。大力发展现代流通业和旅游业，加快发展中介服务业、信息服务业和文化产业，提高商贸和金融服务业发展水平，努力打造现代生产、生活服务平台和高效的公共服务平台。安排1500万元作为服务业发展引导资金，加强对重点行业、关键领域的扶持。积极推动服务业品牌建设，促进服务业对全市经济增长贡献率稳步提高。全面加快保税物流中心建设。

扩大消费需求，发展现代流通。坚持“以工带贸，以贸促工”，以产业集群化推进市场专业化，鼓励专业生产基地向专业生产为基础的商品流通和集散基地发展。积极推进“万村千乡”市场工程建设，大力发展现代商贸业，推进区域性商贸中心和商业网点建设，培育商业龙头企业。通过营造环境，完善政策，加强服务，扶持各类专业市场发展壮大。以社区服务、农村服务为重点，鼓励企业以资本运作、管理技术或品牌效应为纽带，以特许、加盟经营等方式，推进连锁经营。

促进外源型经济由大到既大又强转变。招商引资要从注重出口向注重税收，从轻型产业向适度重型产业，从资金向资金技术人才相结合，从引进为主向消化吸收相结合转变。着力引进基地型项目、高新技术项目和符合产业发展方向、有利于延长产业链、提升产业配套能力的大项目，通过招商选资、“绿色招商”推动产业升级。创新“择大、择优、择强招商”、“产业招商”、“零用地招商”等招商引资方式，扩大农业和服务业招商引资规模。有针对性地引进与我市产业关联度大、带动能力强、发展前景广阔的国内大型企业。做好2006年招商经贸洽谈会各项工作，积极组织参加各类商贸会展。

努力增强经济参与国际竞争的能力。坚持以质取胜和名牌带动，优化对外贸易结构，推动加工贸易转型升级，提高机电产品、高新技术产品和具有自主知识产权、自主品牌产品的出口比重。巩固和扩大传统市场，开拓新兴市场。鼓励有条件的企业“走出去”对外投资和跨国经营，利用境外生产要素资源，带动本市设备和产品出口。充分利用中国-东盟自由贸易区、CEPA以及泛珠三角区域合作平台，加强区域间交流与经贸合作。建立完善反倾销预警机制和贸易壁垒快速联动应对机制。不断提高“大通关”水平。

四、完善城市基础设施体系，提升城市功能

以东部开发为龙头，加快组团发展。以新一轮城市总体规划和概念规划的制定实施为契机，明确城市的功能和定位，彰显特色和优势，加快推进组团发展规划和建设，强化资源整合，统筹产业和基础设施布局。抓好东部组团开发的龙头示范带动作用，发挥多个国家级产业基地和临港优势，积极创建东南绿色工业园。优先建设各组团与中心城区间的快速交通走廊，完善综合服务功能。

加快推进重大功能性城市基础设施建设。充分发挥重点项目对经济发展的拉动作用，建设“四高速五横六纵九加密”公路主干线，构建立体化、区域化、功能化的公路网，加强与周边城市道路网络对接。加快古神公路、福源路、新沙水公路和新岐江公路建设以及麻阳线改造工程。优化中心城区路网结构，加快南、北外环路、长江路大桥和康华路大桥等项目的建设，提高通行能力。加快黄圃、港口、城东等汽车总站，北部、三乡等物流中心及客货运站场建设。实施“公交一盘棋”，加快城北、火炬区等综合站场建设，优化线网布局，不断提高公交智能化水平。配合省做好轻轨、铁路的规划建设。加快中山港二期扩建工程。配合做好嘉明电厂扩建和国电燃气发电厂建设，加快500千伏三乡输变电工程，220千伏青岗站、马鞍站和吉昌站输变电工程等项目规划，争取早日动工。加快建设LNG接收站和城市燃气管网。加快疾控中心、第二人民医院、中心粮库、成品油储备库等工程建设。尽快完成逸仙湖公园、月山公园改造和东明桥公园、紫马岭健身广场建设工程。继续推进危桥涵改造工作，争取完成200座危桥涵改造任务。

管理和配置好城市资源。积极寻求资源配置在城市容量、秩序、结构、规模等方面的效益最大化和最优化，盘活城市公共资源和无形资产。充分发挥规划对城市资源的调控作用，做好新一轮土地利用总体规划修编、旧城区改造和风貌保护工作。严格控制并开发好城市建设用地，加大土地储备力度。在市属行政公益性项目中加快推行代建制，加快城市道路、市政公用事业的市场化改革。进一步完善城管执法运行机制，坚持专项整治与长效管理并举，切实解决好流动摊档、城市“牛皮癣”、违章建筑、噪音污染等群众反映强烈的问题。

五、坚持“科教兴市”战略，全面提升城市文明水平

加快科技创新步伐。按照建设“创新型中山”的要求，完善城市创新体系建设。坚持引进与创新相结合，走引进、消化、吸收、创新之路，促进产学研相结合，推进“科技兴镇”试点工作。加强对先进制造业、现代农业、现代服务业、社会发展和节能环保五个重点领域的自主创新。加强专业镇或特色产业基地的公共技术创新平台、实验室建设，支持条件成熟的镇区建设自主创新基地和成果转化孵化器，鼓励大中型企业组建研发机构。安排科技三项经费1900万元，进一步完善政府引导资金的投入、使用和监管机制。设立市级风险投资基金，鼓励发明创造，推动专利技术产业化。加大知识产权保护力度。

坚持教育优先发展，努力推进教育现代化。全面推进基础教育均衡化、优质化，逐步实现高标准、高质量义务教育，今年秋季起在农村实行免费义务教育。进一步落实振兴初中行动计划，加强示范性高中建设。规范发展幼儿教育，积极发展高等教育、成人教育，合理布局、超常规发展高（中）等职业技术教育。鼓励民办教育发展，积极推进现代农民教育工程。继续推进“三名工程”，着力提高教师队伍的整体素质，促进教育内涵式发展。加快市职业技术学院建设。积极推进学习型城市建设，构建终身教育体系。

推进人才资源开发，为经济发展提供智力保障。加强产业发展急需的各类人才的引进和培养，完善人才集体户和公共户管理体制，畅通人才入户渠道，为人才稳定就业和创业营造良好的政策环境。加强对重点产业和行业人才，尤其是高技能人才的培养、引进、储备和使用。加强博士后流动站建设，着力建设一支自主创新型的领军人物和科研团队。切实加强公务员、企业经营管理、专业技术及技能型人才队伍建设，加快人才集聚。

发展文化事业，建设文化强市。积极开展历史文化资源的发掘保护和开发利用，加快翠亨村国家历史文化名村规划和申报工作。加快文化基础设施建设，充分发挥市文化艺术中心、商业博物馆等阵地的作用，丰富广大市民的精神文化生活。积极开展民间、民俗文化的传承和发展工作。着力推进文化产业发展，积极筹办南朗、小榄文化产业园区。加快广播电视新闻出版事业的改革与发展。

六、深入推进各项改革，增创体制机制新优势

深化行政管理体制和事业单位改革。推进政企分开、政资分开、政事分开、政府与市场中介组织分开。坚持总体规划、分类指导、配套改革、稳妥推进的原则，根据事业单位的不同性质和功能，进行事业单位改革，做好精编减负工作。积极推进科技、教育、文化、体育、卫生等各项改革。

不断完善国有资产管理体制。积极探索履行出资人职能的有效方式，稳步推进国有企业产权多元化。统筹定位、分类指导国有企业发展，确保国有资产保值增值。加快建立国有资本经营预算制度，建立健全经营性资产、非经营性资产、自然资源资产等监管体制，坚持财务总监制度，防止国有资产流失。

推进财税金融体制改革。加快完善公共财政体制，深化预算决算改革。完善转移支付体制和市镇分税分成体制。加强税收管理。进一步加强和规范非税收入管理。深入推进财政支出绩效评价工作。深化投资体制改革，鼓励多种投资主体参与基础设施项目投资、建设、管理和运营。积极发展资本市场，做好中小金融机构处置工作。加强金融生态建设，维护金融稳定和安全。

标本兼治，规范市场经济秩序。继续加大力度整顿市场经济秩序，严打侵权、商业欺诈、偷逃骗税等违法行为。抓好食品、药品安全的监管和专项整治，深入开展无证照经营查处取缔行动。严厉打击传销和变相传销。加强对粮食、燃油、燃气等重要商品的市场监管。

七、坚持发展与资源环境人口相协调，促进人与自然的和谐

大力发展循环经济。按照建设资源节约型和环境友好型社会的要求，加快生态市创建步伐。积极发展生态农业、生态工业、生态旅游服务业和环保产业，加强废旧物资回收管理，推动再生资源产业发展，努力构建循环经济型产业体系。加快制定行业节能降耗的标准和措施，积极推广资源综合利用的新技术、新工艺和新设备，鼓励和推动企业以节能降耗为核心的技术改造。严格环保准入要求，加快建设生态园区，构建生态产业链。加强用水管理，完善水价机制。倡导循环消费，增强全社会的节约意识。

加强生态保护，建设“绿色中山”。积极创建“广东省林业生态市”，安排4000万元用于“绿化中山大地工程”，中心城区新增公共绿地面积17.8公顷，绿地率达到36%。积极开展创建绿色学校、绿色家庭、绿色社区、绿色企业等活动，鼓励创建生态镇、村和社区。推动重要生态功能区建设，加强水土流失综合治理、湿地保护和生态公益林建设，初步建立起城乡一体化生态安全保障体系。保护山体资源，加强采石场整治关闭和复绿工作。重点抓好岐江河综合整治工程、镇区生活污水处理厂建设工程、生活垃圾综合处理基地建设、危险废物安全处置、林相改造建设工程、五桂山生态保护区建设、沿海红树林培育、绿色无公害农产品生产示范基地建设、农产品监测网络工程和工业污染源监控系统建设等10个项目。

加大执法力度，有效防控污染。强化环保责任制，全面加强重点区域流域环境综合治理，着力解决严重威胁人民群众健康安全、严重影响区域投资环境的环境污染问题。实施污染源分级管理，削减排污总量。加快北部、南部组团垃圾综合处理基地及镇区垃圾转运站建设。继续实施“碧水工程”。完成中嘉污水处理厂二期建设，加快推进珍家山污水处理厂首期污水收集系统工程，抓好崩山涌等7条河涌整治。推进“供水一盘棋”，加强8个饮用水源保护。健全环境监管长效机制，提高固体废物资源化与无害化处置率，加强酸雨污染防治和机动车尾气污染治理。积极推进农村污染防治，控制农业面源污染。

八、构建“和谐中山”，着力解决群众最关心、最现实的切身利益问题

统筹城乡就业，构建覆盖面更广、保障水平更高的社保体系。坚持发展为了人民，发展依靠人民，发展成果由人民共享。完善公共就业服务体系，加快建设统一的、覆盖城乡的劳动力市场。安排1200万元用于促进就业专项资金，培训农村富余劳动力3万人，加快农村富余劳动力向非农产业转移，帮助“4050”人员和“零就业家庭”就业。逐步探索城乡社会保险一体化，实现全市社会保险参保达350万人次以上，切实提高保障水平。加快推进农村基本养老保险工作，年内新增10万人次参保。全面实施新型农村合作医疗镇区统筹，探索新型农村合作医疗向医疗保险过渡。按照“一镇一所敬老院”的目标，全面加快发展农村养老事业，实施好城乡孤寡老人集中供养工程。加强低保动态管理。完善社会救助体系，安排964万元用于农村救济，动员全社会力量发展慈善事业。实施旧城改造和城市“双困户”住房建设计划，建设“双困户”住房2.3万平方米。做好扶困助学工作。加强法律援助和司法救助工作。

改善医疗卫生条件，提高市民健康水平。继续完善公共卫生体系建设，提高疾病预防控制能力、卫生执法监督能力和突发事件应急处理能力，逐步建立覆盖市、镇、村（社区）公共卫生医疗急救信息系统。规范医疗市场，形成比较完善的市、镇两级卫生执法监督体系。加强农村卫生工作，发展社区卫生服务，着力解决群众看病难、看病贵问题，逐步满足群众不同层次的医疗保健需求，继续推行“济困助医”活动。加强对艾滋病等重点传染病的预防控制工作，深入开展爱国卫生运动，巩固创建卫生镇成果。

努力建设“富裕、公平、活力、安康”的社会环境。以构建“和谐中山”为目标，大力推进社会主义精神文明和政治文明建设，着力推进现代公民素质教育，深入开展群众性精神文明创建活动，推进全社会德育体系建设。统筹协调利益关系，注重社会公正和公平，重视解决不同阶层收入差距过大问题，不断提高各阶层居民的收入。深入推进全民健身运动，实现所有镇区建有全民健身广场，80%以上行政村（社区）建有健身苑点，继续提高竞技体育水平。扎实做好计划生育工作，稳定人口低生育水平，不断提高人口素质。加强国防教育，扎实做好“双拥”工作，努力做好老龄、残疾人等各项工作，大力推进妇女、儿童事业发展规划的实施。

确保社会稳定。积极应对矛盾凸显期的社会治安挑战，进一步建立健全社会治安防控体系。加强综合治理，建设平安和谐社区，加强外来流动人口及出租屋管理，依法严厉打击各种犯罪活动。建立健全利益协调机制，畅通诉求渠道，做好信访和人民调解工作。积极预防和妥善处置群体性突发事件。认真吸取“12·25”特大火灾事故教训，以铁的手腕抓好安全生产，健全监管体制，深入排查事故隐患，铲除事故温床。加强交通安全监管。加强自然灾害预测预报工作，提高防灾减灾能力。

努力建设人民群众满意的政府

全面推进依法行政。继续落实《全面推进依法行政实施纲要》，推进依法治市的各项建设。按照精简、统一、效能的原则和决策、执行、监督相协调的要求，规范部门职能。切实做到依法决策、依法办事、依法处理问题。不断完善权责明确、行为规范、监督有效、保障有力的行政执法体制。认真执行《广东省政务公开条例》，健全行政执法责任制、过错责任追究制，加大对违法和行政不作为的问责力度。

推动科学、民主行政。坚持市政府组成人员与人大常委会、政协委员座谈制度，及时办理人大议案、建议和政协提案。进一步健全民主制度，丰富民主形式，创造条件让社会组织、企业和广大市民更广泛更直接地参与社会公共事务管理。坚持“问计于民”，继续扩大政务公开范围，健全公开征求意见、专家咨询论证和决策听证等制度。继续办好《政报》，完善规范性文件公开发布和政府新闻发布工作，提高政府公信力和群众对政府工作的满意度。

增强履行职能的能力。切实把政府经济管理职能转到主要为市场主体服务和创造良好发展环境上来。巩固和扩大行政审批制度改革成果，按照“整合、共享、务实、便民、高效、节约”的原则，继续推进电子政务建设，不断提高行政效率。大兴求真务实之风，加强调查研究，提高决策的前瞻性和可操作性。建立健全各种预警和应急机制，提高政府应对重大突发事件和风险的能力。减少一般性事务性活动，精简会议、文件，降低行政运作成本。努力提高公务员素质、创新能力和工作水平。

打造清正廉洁、干净干事的公务员队伍。以《公务员法》的贯彻实施规范公务员队伍建设。全体公务员要坚持“两个务必”，不断深入推进先进性教育，牢固树立为民、务实、清廉观念。认真贯彻中共中央《建立健全教育、制度、监督并重的惩治和预防腐败体系实施纲要》，继续保持惩处腐败的高压态势，规范和完善建设工程招投标、经营性土地使用权出让、产权交易和政府采购等各项制度。

各位代表，“十五”伟业完成了辉煌的使命，“十一五”宏图踏上了崭新的征程。展望中山美好家园的壮丽前景，我们既信心百倍，豪情满怀，更感责任和使命重大。让我们紧密团结在以胡锦涛同志为总书记的党中央周围，高举邓小平理论伟大旗帜，以“三个代表”重要思想为指导，牢固树立和落实科学发展观，深入贯彻党的十六大和十六届五中全会精神，在省委、省政府和市委的领导下，坚定信心，振奋精神，励精图治，为推动我市经济社会发展的新跨越，建设“两个适宜”的美好家园，构建和谐中山，实现富裕安康而努力奋斗！
